# An auditory-visual cooperative perception method for honking vehicle localization

**DOI:** 10.1371/journal.pone.0337352

**Published:** 2025-11-21

**Authors:** Fei Yuan, Junxi Kang, Jiao Yin, Jinli Cao

**Affiliations:** 1 School of Automation, Guangdong Polytechnic Normal University, Guangzhou, Guangdong, China; 2 School of Automation, Guangdong University of Technology, Guangzhou, Guangdong, China; 3 Department of Computer Science and Computer Engineering, La Trobe University, Melbourne, Victoria, Australia; VIT Bhopal University, INDIA

## Abstract

Locating honking vehicles is crucial for controlling arbitrary honking and reducing environmental noise. However, traditional methods for honking vehicle localization, which utilize sound source localization technology, suffer from inaccuracies and limited detection range due to the multipath effects of sound propagation and environmental noise interference. To address these challenges, an auditory-visual cooperative perception (AVCP) method for honking vehicle localization is proposed, and a detailed workflow of this method is presented. In the AVCP method workflow, the Emphasized Channel Attention, Propagation, and Aggregation in Time-Delay Neural Network (ECAPA-TDNN) is used to recognize honking vehicle models from captured audio signals, as different vehicle models exhibit distinct horn sound characteristics. Subsequently, YOLO v9 is employed to detect vehicles and recognize their corresponding models in the images captured by the camera. Thus, among the vehicles detected and identified using YOLO v9, the honking vehicle is determined as the one whose model matches the vehicle model recognized by ECAPA-TDNN. Additionally, experiments with simulated and public datasets were conducted to evaluate the performance of the AVCP method for honking vehicle localization. The experimental results show that the AVCP method is less susceptible to environmental noise and can more accurately identify and locate vehicles from greater distances compared to traditional methods based on sound source localization technology.

## Introduction

With the continuous growth of the automobile industry, the global vehicle population has been steadily increasing, encompassing a wide range of models including trucks, cars, and other vehicles. While this expansion has significantly enhanced the efficiency of goods transportation and facilitated personal mobility, it has also contributed to widespread traffic congestion, particularly in urban settings. In such congested conditions, irresponsible drivers often resort to excessive honking, exacerbating noise pollution. This issue is especially pronounced in densely populated urban areas and sensitive locations such as schools and hospitals, where excessive noise can have profound health implications [1–3].

Emerging evidence highlights the adverse health impacts of transportation-related noise, particularly from road traffic. Chronic exposure has been linked to increased cardiovascular morbidity and mortality through mechanisms such as sleep fragmentation, elevated stress hormone levels, and oxidative stress, all of which contribute to vascular dysfunction and hypertension [4]. According to estimates by the World Health Organization, traffic noise accounts for over 1.6 million healthy life-years lost annually in Western Europe. Supporting this, a large-scale UK Biobank study involving more than 370,000 participants reported that noise levels exceeding 65 dB(A) are significantly associated with elevated blood pressure, triglyceride levels, and glycated hemoglobin, independent of air pollution exposure. Additionally, excessive noise has been shown to exacerbate anxiety and stress, underscoring the urgent need for effective noise mitigation strategies [5].

Beyond cardiovascular health, excessive noise from honking can heighten anxiety and stress levels among drivers and pedestrians alike, potentially leading to broader psychological and social consequences [6,7]. Given these multifaceted impacts, implementing effective measures to monitor and mitigate vehicle honking is imperative to reduce arbitrary noise pollution and safeguard public health.

There are three primary approaches to controlling vehicle honking. The first involves placing no-honking notice boards along roadsides, serving as reminders for drivers to use their vehicle horns responsibly. The second approach entails developing a honking control system that can regulate honking behavior, such as calculating the number of honks within a certain period to alert drivers [8] or automatically adjust to a low-volume horn in silent zones [9]. The third approach involves the installation of a honking monitoring system that enables the identification of vehicles engaged in honking [10,11]. While the first approach lacks enforcement, making it less effective, and the second approach proves impractical and costly, the third approach is more efficient as it actively detects honking vehicles in noise-sensitive areas.

The pivotal technologies within the honking monitoring system are sound recognition and sound source localization (SSL), which utilize a microphone array to capture multi-channel sound signals. The sound signals undergo processing using sound recognition technology to identify the presence of a honking sound. Upon successful identification of honking sounds, its location is determined by applying SSL techniques. Specifically, SSL techniques estimate the direction and distance of a sound source relative to a microphone array by calculating parameters such as the Direction of Arrival (DOA) and source range [12,13]. Currently, three predominant approaches is included within SSL research area: Time Difference of Arrival(TDOA) [14,15], Beamforming algorithms [16,17], and High-Resolution Spectral Estimation techniques [18].

The TDOA method estimates the source position by analyzing temporal differences in the arrival times of acoustic signals across spatially distributed microphones. While computationally efficient, it is notably susceptible to environmental reverberation. Beamforming techniques implement spatial filtering by coherently combining multi-channel signals, enabling real-time directional enhancement primarily through delay-and-sum operations. However, their spatial resolution is inherently constrained by the physical dimensions, or aperture, of the microphone array. High-resolution spectral estimation methods, such as MUSIC, rely on eigen-based subspace decomposition, effectively separating signal and noise components through their inherent orthogonality. However, the accuracy of these methods strongly depends on the precision of microphone array configurations and sensor calibration; even minor deviations can significantly degrade their performance. Furthermore, localization effectiveness substantially deteriorates under low signal-to-noise ratio (SNR) conditions, reducing reliability in noisy environments.

Although SSL technology has been extensively studied and successfully applied in various research areas, such as sound source separation [19,20], automatic speech recognition [21,22], speech enhancement [23,24], and human-robot interaction [25,26], its localization effectiveness remains significantly influenced by multipath effects of sound propagation and environmental noise interference. These limitations are particularly pronounced in open and dynamically changing outdoor environments, restricting the practical applicability of SSL technologies. In studies addressing the localization of honking vehicles, researchers have commonly employed conventional SSL technologies implemented through meticulously designed microphone arrays [10,11]. The honking monitoring system was designed using a 3D microphone structure in a spiral distribution [10]. Based on the far-field model, the Time Difference of Arrival (TDOA) is calculated using Generalized Cross-Correlation (GCC) algorithm to estimate the direction of the sound source. Experimental results demonstrate that localization is accurate within distance of less than 25 meters. However, the localization accuracy decreases notably as the distance increases within the range of 25 to 40 meters. In [11], a planar array of 32 MEMS microphones was designed to implement the steered response power phase transform (SRP-PHAT) localization algorithm, supporting the localization and tracking of vehicles. Observations indicated that the effectiveness of localization remains nearly 100% at distances of 5 meters and 10 meters on the x-axis, decreasing with greater distances.

Obviously, traditional honking vehicle localization methods based on SSL technology become increasingly inaccurate as the distance increases due to the greater multipath effects of sound propagation and environmental noise interference over larger distances. To expand the detection range of honking vehicles, this paper reports an auditory-visual cooperative perception (AVCP) method for honking vehicle localization, accompanied by a comprehensive elucidation of its workflow.

Given the unique honking sound characteristics exhibited by different vehicle models, the Emphasized Channel Attention, Propagation, and Aggregation in Time-Delay Neural Network (ECAPA-TDNN) is employed to discern the model of honking vehicle from collected honk sound signals. Subsequently, YOLO v9 is utilized to detect all vehicles and identify each vehicle’s model in the captured image, benefiting from its rapid computation and high accuracy. Hence, among the vehicles detected and identified through YOLO v9, the honking vehicle is identified as the one whose model corresponds to the vehicle model recognized by ECAPA-TDNN. To evaluate the performance of the proposed method, experiments are carried out under different scenarios, and several key factors that influence the performance of the proposed method are analyzed based on simulated and public datasets.

Compared to traditional honking vehicle localization methods based on SSL technology, the AVCP method proposed in this paper does not rely on acoustic propagation characteristics, such as signal arrival times or directional angles. Instead, it exclusively exploits the acoustic fingerprint features inherent in honking sounds, which are identified using the ECAPA-TDNN model. This approach effectively mitigates localization errors caused by multipath propagation and environmental noise interference. Additionally, by integrating vehicle appearance recognition through YOLOv9, the proposed method precisely determines vehicle positions, thereby overcoming inaccuracies commonly associated with traditional SSL-based honking vehicle localization techniques.

The remainder of this paper is organized as follows. Section 2 presents the workflow of the AVCP method for honking vehicle localization in detail. In Section 3, comprehensive performance evaluation experiments are presented with simulated and public datasets, including the impact of background noise of different intensities, the impact of audio signal input modes and the impact of distance between vehicles and capturing camera. Section 4 presents the conclusion of this study and our future work. In summary, the main contributions of our work are as follows.

1. An auditory-visual cooperative perception (AVCP) method for honking vehicle localization is proposed to address the drawbacks in existing methods that rely solely on SSL technology, which are susceptible to environmental noise and cannot accurately identify and locate honking vehicles from greater distances.
2. A detailed workflow of the AVCP method for honking vehicle localization is presented. With the constructed workflow, the honking vehicle’s model and location can be accurately determined in the captured image. Moreover, state-of-the-art audio or image recognition methods can be conveniently applied in the proposed workflow to obtain more accurate performance.
3. For a real-world honking vehicle monitoring system employing the AVCP method, performance evaluation can be conveniently conducted using the designed performance evaluation experiments specific to the AVCP method for honking vehicle localization.

## Methodology

**Fig 1:**
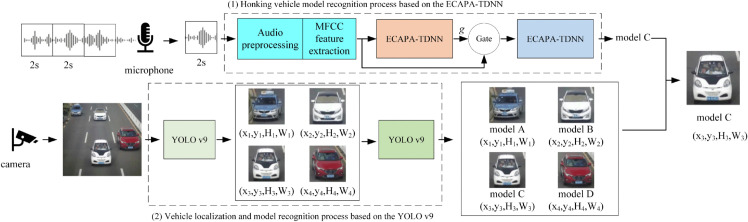
Workflow of the AVCP method for honking vehicle localization.

The methodology presented in this paper comprises two key components: the honking vehicle model recognition process based on ECAPA-TDNN and the vehicle localization and model recognition process based on YOLO v9. The workflow of the AVCP method for honking vehicle localization is illustrated in Fig 1. As depicted in Fig 1, the audio signal captured by microphone is segmented into 2-second digital audio files. Each 2-second audio file serves as the input for the honking vehicle model recognition process based on ECAPA-TDNN, with the model of the honking vehicle being the output of this recognition process.

While the microphone preserves a 2-second digital audio file, the camera captures the traffic scene image and inputs it into the vehicle localization and model recognition process based on YOLO V9. The output of this process includes the locations of the vehicles and their corresponding models.

It is then straightforward to determine that, among the vehicles identified and located with YOLO V9, the honking vehicle is the one whose model matches the vehicle model recognized by ECAPA-TDNN.

In the following two subsections, the honking vehicle model recognition process based on ECAPA-TDNN and the vehicle localization and model recognition process based on YOLO V9 are presented in detail.

### Vehicle model recognition process based on ECAPA-TDNN

Benefiting from the distinctive sound characteristics and timbre displayed by vehicle horns of different models, the use of an ECAPA-TDNN neural network enables the classification of the spectral features found in the horn signals of vehicle models. Numerous features facilitating honking vehicle model recognition are located in the high-frequency band, which undergoes rapid decay during transmission. Therefore, amplifying the energy of the high-frequency component of the audio signal and extracting an effective feature representation becomes crucial for honking vehicle model identification.

As shown in Fig 1, the 2-second digital audio file is preprocessed to enhance the high-frequency band, and Mel-frequency cepstral coefficients (MFCC) are extracted to obtain feature vectors for improved separability and recognizability.

#### Audio signal preprocessing.

**Fig 2:**
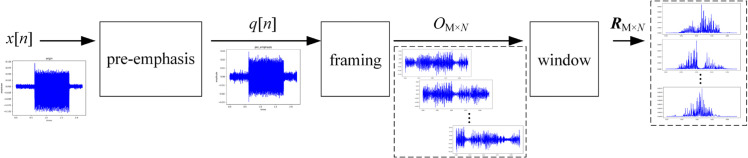
The flowchart of audio signal preprocessing.

To enhance the high-frequency band contained in the audio signal, a preprocessing operation is conducted on the 2-second audio file before extracting MFCC features, as depicted in Fig 2. In Fig 2, the first step of audio signal preprocessing is pre-emphasis, which is designed to filter the audio signal to amplify its high-frequency components. This procedure mitigates potential high-frequency signal attenuation and distortion during recording and transmission, thereby improving the overall audio signal quality and its high-frequency resolution. Typically, the pre-emphasis filter is configured as a first-order digital filter. The resulting q[n] is calculated according to (1).

q[n]=x[n]−αx[n−1]. (1)

Where *α* represents the filter coefficient, typically ranging from 0.9 to 1. Commonly utilized values for *α* are 0.95 or 0.97.

The second step of audio signal preprocessing involves framing. Framing divides the signal into short-time windows to facilitate feature extraction within each window. The typical window length ranges from 20 to 40 milliseconds, with more than 50% overlap between adjacent windows to maintain signal continuity. As illustrated in Fig 2, *q*[*n*] is subdivided into M frames, each comprising N samples, thereby creating a matrix of ${\text{O}}_{M\times N}$.

After framing, each frame in ${\text{O}}_{M\times N}$ undergoes windowing to emphasize the sampled part while attenuating the remainder. The Hamming window is a commonly used windowing function, known for its favorable pass-band characteristics, ability to reduce spectral leakage, and suitability for smoothing short-duration signals. The detailed equation for the Hamming window function *W*(*n*) is provided as follows.

$$W(n)=0.54-0.46\cos(\frac{2n\pi }{N-1}).$$ (2)

where *n* is a positive integer not larger than N.

During the audio signal preprocessing, the original 2-second audio signal is transformed into a multi-frame digital signal, represented by the matrix ${\text{R}}_{M\times N}$ in Fig 2. This transformed signal serves as the input for the MFCC feature extraction.

#### MFCC features extraction.

**Fig 3:**
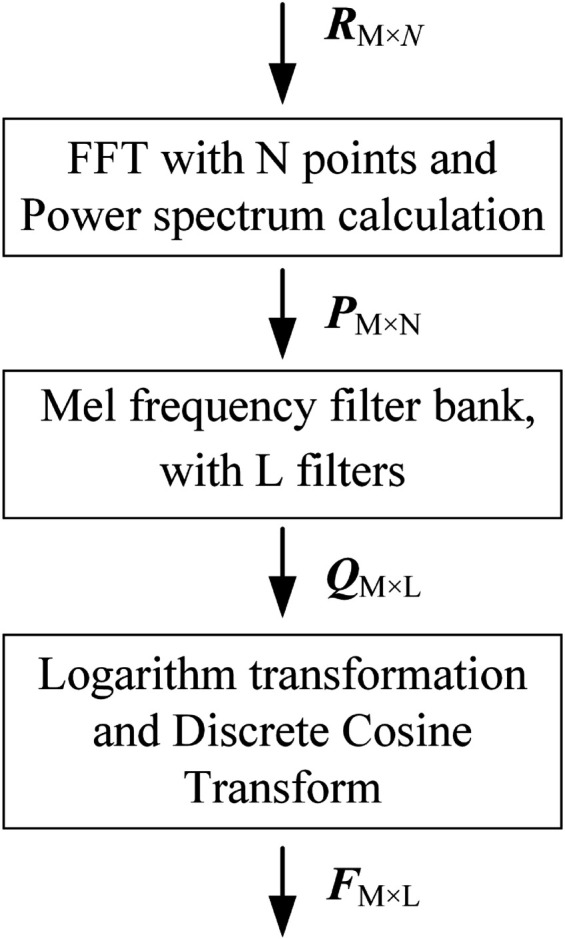
Flow chart of MFCC feature extraction.

MFCC is designed based on the knowledge of the human auditory system. It is a set of features comparable to chroma or spectral features. Fig 3 demonstrates the process of MFCC feature extraction.

Firstly, each frame in ${\text{R}}_{M\times N}$ undergoes a Fast Fourier Transform (FFT) to be converted into a frequency domain signal. The FFT of the *i*-th frame, denoted as $r_{i}(\text{n})$, in ${\text{R}}_{M\times N}$ and having a length of N is calculated using (3).

$$R_{i}(k)=\sum_{n=0}^{N-1}r_{i}(n)e^{-j\frac{2\pi }{N}nk},0\leq k\leq N-1.$$ (3)

Meanwhile, the power spectrum of $R_{i}(\text{k})$ is obtained using (4), and is used to form the matrix ${\text{P}}_{M\times N}$, as depicted in Fig 3.

$$P_{i}(k)=\frac{|R_{i}(k){|}^{2}}{N},\;0\leq k\leq N-1.$$ (4)

To obtain the Mel spectrum of the *i*-th frame, the power spectrum $P_{i}(\text{k})$ is convolved with a set of Mel-scale triangular filter banks. The convolution of $P_{i}(\text{k})$ with each filter, $H_{l}(\text{k})$, is computed at every frequency. Let us define a triangular filter bank comprising L filters. The frequency response of each triangular filter, $H_{l}(\text{k})$, is calculated using (5), resulting in the Mel filter bank ${\text{H}}_{L\times N}$.

$$H_{l}(k)=\left\{ \begin{array}{cc} 0, & \;k<f(l-1)\\ \frac{k-f(l-1)}{f(l)-f(l-1)}, & \;f(l-1)\leq k\leq f(l)\\ \frac{f(l+1)-k}{f(l+1)-f(l)}, & \;f(l)\leq k\leq f(l+1)\\ 0, & \;k>f(l+1) \end{array}\right..$$ (5)

Where *f*(*l*) represents the center frequency of the Mel triangular filter, which is correlated with the number of Mel filters. Consequently, the resultant matrix ${\text{Q}}_{M\times L}$ is obtained according to (6). Namely, it is the result of the power spectrum ${\text{P}}_{M\times N}$ passes through the Mel filter bank ${\text{H}}_{L\times N}$.

$$Q_{M\times L}=P_{M\times N}\cdot H_{L\times N}^{T}.$$ (6)

Subsequently, the next step involves performing a logarithmic operation followed by a Discrete Cosine Transform (DCT), as expressed by (7). This process yields what are referred to as MFCC features.

$$f(i,l)=\sum_{m=1}^{L}\ln q_{i}(m)\cdot \cos\left\lfloor \frac{\pi l(2m-1)}{2M}\right\rfloor ,1\leq i\leq M,1\leq l\leq L.$$ (7)

Where M represents the number of frames, and L denotes the number of filters in the Mel filter banks.

The obtained MFCC features, denoted as ${\text{F}}_{M\times L}$, serve as the input to the ECAPA-TDNN, which is employed to detect the presence of honking sounds within the audio signal. If a honking sound is detected, another ECAPA-TDNN is used to identify the corresponding vehicle model. Therefore, the following subsection will introduce the ECAPA-TDNN.

#### ECAPA-TDNN for honk sound detection and vehicle model recognition.

**Fig 4:**
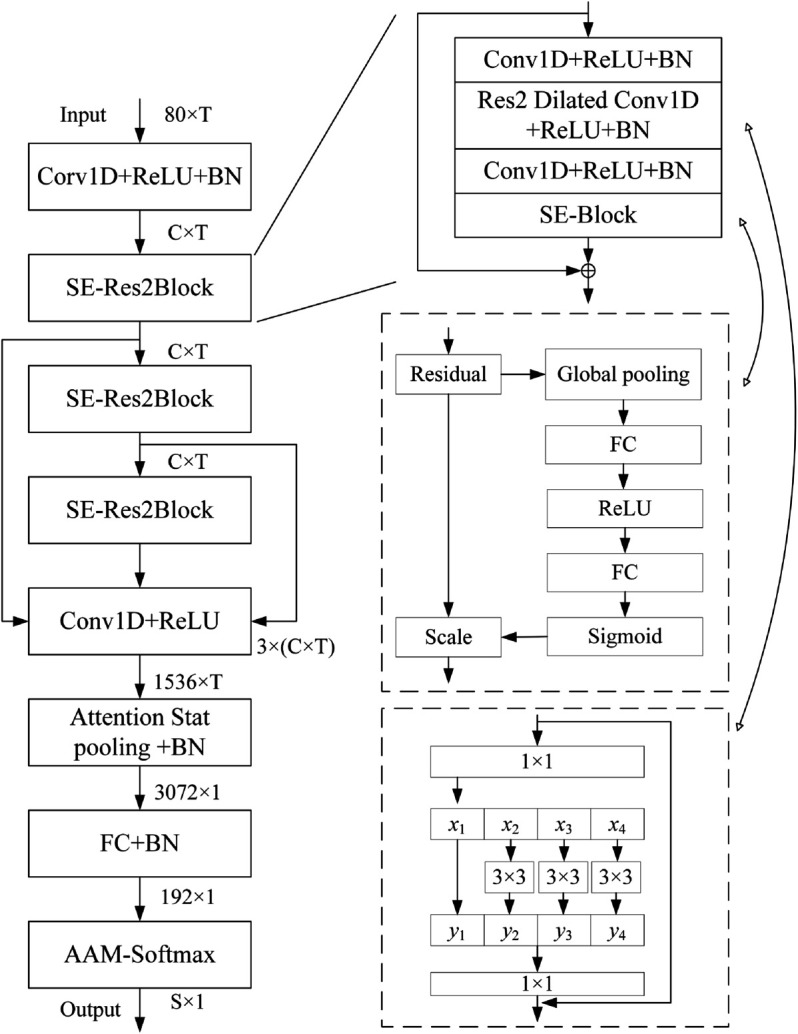
Network of the ECAPA-TDNN.

The ECAPA-TDNN is a time-delay neural network model that has demonstrated remarkable performance on the Vox Celeb public dataset. This network architecture is specifically designed for sound recognition tasks, accounting for both the time and frequency domain characteristics as well as the complex feature composition of audio signals. The ECAPA-TDNN aims to achieve precise and swift sound recognition across diverse scenarios by incorporating methodologies such as channel attention, residual connections, and multi-layer feature aggregation. The network structure of the ECAPA-TDNN [27] is illustrated in Fig 4.

The ECAPA-TDNN takes MFCC feature vectors as input and processes them through a series of TDNN layers, each with a distinct time context range, enabling the capture of audio information at different scales. The key to achieving honking vehicle model recognition lies in the model’s ability to statistically pool audio information of varying scales through the ASP layer and enhance channel attention using SE blocks. Subsequently, a fully connected layer and a Softmax layer are applied to convert the output into a probability distribution, which is then mapped to the target vehicle category, resulting in the recognition of the honking vehicle model.

**Fig 5:**
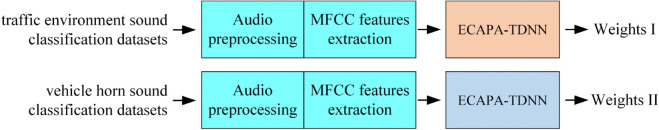
Training process for both ECAPA-TDNN.

In the proposed workflow, depicted in Fig 1, two ECAPA-TDNN models with identical structures are trained. The first ECAPA-TDNN is trained using traffic environment sound classification datasets. After this training, it is utilized to detect the presence of horn sounds within the audio signal. The second ECAPA-TDNN is trained using vehicle horn sound classification datasets, enabling the identification of the associated vehicle model. The training process for both ECAPA-TDNN models is depicted in Fig 5. As depicted in Fig 5, the MFCC features within the training data need to be extracted before being input into the ECAPA-TDNN models. This process is elaborated on in the previous two subsections. After the training process, the two ECAPA-TDNN models and their corresponding weights, denoted as Weight I and Weight II in Fig 5, can be acquired for the purposes of honking sound identification and vehicle model recognition.

Meanwhile, the localization of the honking vehicle also requires determination through visual recognition methods. Therefore, the proposed AVCP method for honking vehicle localization utilizes YOLO V9 for vehicle localization and vehicle model recognition in captured images. The following subsection will provide an introduction to the vehicle localization and model recognition process based on the YOLO V9.

### Vehicle localization and model recognition process based on the YOLO V9

In Fig 1, two YOLO V9 networks are included in the vehicle localization and model recognition process. The first YOLO V9 network is used to recognize and locate all vehicles in the captured image. Then, the second YOLO V9 network is used to recognize each localized vehicle’s model. The first YOLO V9 network utilizes pre-trained weights derived from the COCO dataset, which encompasses 80 categories. The second YOLO V9 network is pre-trained with vehicle model classification datasets, such as the VRID datasets utilized in this study.

With these two trained YOLO V9 networks, the initial YOLO V9 generates multiple bounding boxes for vehicles, each defined by the *x* and *y* coordinates of the box center, along with its width and height. Subsequently, the input image is cropped based on the resulting bounding boxes. These cropped images are then fed into the secondary YOLO V9 network. The secondary YOLO V9 subsequently assigns a label and confidence value to each cropped image, accurately identifying the model of the vehicle. Finally, the position of the honking vehicle is pinpointed as the one whose model corresponds to the identified model recognized from the captured audio signal as depicted in Fig 1.

**Fig 6:**
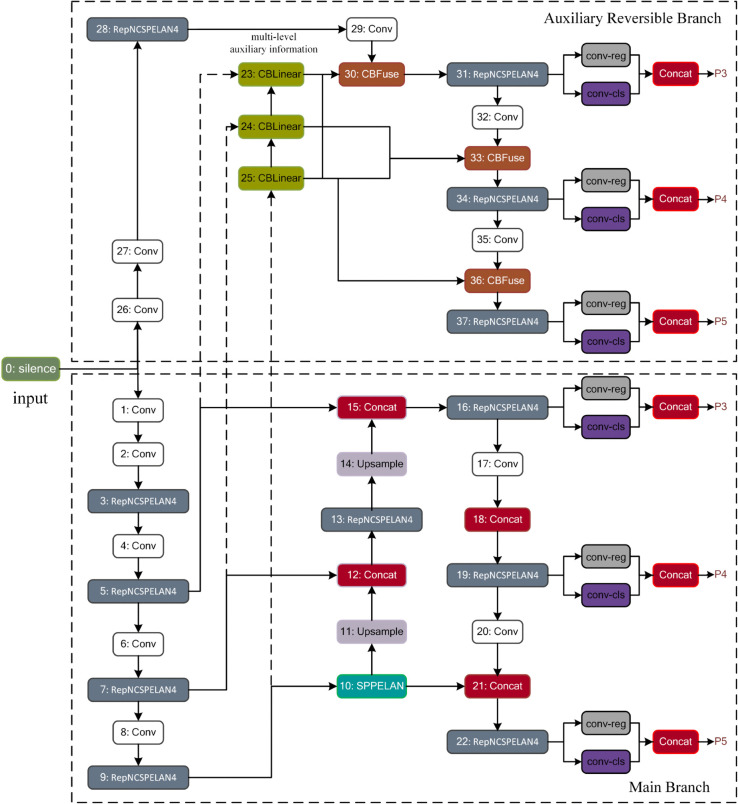
Network structure of the YOLO V9.

The utilized YOLO V9 network builds upon the foundations of the YOLO v7 network, introducing several enhancements to improve performance. A notable innovation in YOLO V9 is the implementation of the Generalized Efficient Layer Aggregation Network (GELAN). GELAN melds features from two distinct neural network architectures: Cross Stage Partial Network (CSP Net) and Efficient Layer Aggregation Net (ELAN), creating a robust framework that prioritizes lightweight design, inference speed, and accuracy. Additionally, YOLO V9 incorporates Programmable Gradient Information (PGI), designed to mitigate the information loss that occurs as network depth increases. This approach allows for more effective learning processes and enhanced error back-propagation through deeper network structures. The synergistic integration of PGI and GELAN in YOLO V9 yields significant performance advancements, positioning it well above existing real-time object detectors across various metrics on the MS COCO dataset. Fig 6 illustrates the network structure of YOLO V9 [28].

## Experimental

The proposed AVCP method for honking vehicle localization comprises two main processes: the vehicle model recognition process based on ECAPA-TDNN and the vehicle localization and model recognition process based on YOLO V9. Consequently, the experiments in this section focus on assessing the recognition accuracy of each process. Before conducting the performance evaluation experiments, an introduction to the experimental configuration, the audio and image datasets used for training and testing, and the performance evaluation metrics is provided.

### Experimental configuration

The hardware configuration for the evaluation experiments includes an Intel(R) Core(TM) i7-13700 CPU, NVIDIA GeForce RTX 4070 GPU, and 12GB RAM. The software environment comprises Windows 10, Python 3.8, CUDA 10.1, and the deep learning framework Pytorch 1.8.1+cu101.

In the audio signal preprocessing stage, the microphone’s sampling frequency is set to 22,050 Hz. The configuration involves framing each 2-second audio signal into frames of 134 counts, each frame consisting of 1024 sample points, with a frame shift of 320 points. For the FFT, a window size of 1024 is utilized, and the Mel filter banks encompass 40 filters. Consequently, the dimension of the MFCC features matrix is 134×40.

Regarding the ECAPA-TDNN configuration, the batch size is 16, utilizing the Adam optimizer with an initial learning rate of 0.001. The weight decay coefficient is set to 10^−6^, and the training process spans 30 epochs.

### Audio and image datasets

#### Vehicle horn sound classification datasets.

In the pursuit of training the ECAPA-TDNN for the identification of specific vehicle models based on their emitted horn sounds, a comprehensive vehicle horn sound classification dataset was compiled using FORZA HORIZON 4, a vehicle honk sound simulation software. The simulated dataset consists of 800 audio signals for each distinct vehicle model, categorized into four groups: 200 honking audio samples free from extraneous noise, 200 samples overlaid with white noise, 200 samples accompanied by simulated wind and rain sounds, and 200 samples with controlled pitch and tempo modifications. Each honking audio sample in the dataset maintains a consistent duration of two seconds.

#### Traffic environment sound classification datasets.

To utilize the ECAPA-TDNN model for vehicle honking sound detection within audio signals, a comprehensive traffic environment sound classification dataset has been meticulously constructed. This dataset includes two distinct classes: “audio signal with honk sound” and “audio signal without honk sound”.

The “audio signal without honk sound” class is methodically assembled by excluding the sound category related to vehicle horn emissions from both the UrbanSound8K dataset [29] and the Acoustic Event dataset [30]. The UrbanSound8K dataset comprises 8,732 audio samples spanning 10 urban sound categories, while the Acoustic Event dataset encompasses 5,223 audio samples covering 28 sound categories, such as engine noise, footsteps, and speaker sounds, etc.

Conversely, the “audio signal with honk sound” class comprises 8,000 audio samples, as detailed in the previous dataset description of the “Vehicle Horn Sound Classification Dataset.”

This construction of the traffic environment sound classification dataset facilitates a rigorous evaluation of the ECAPA-TDNN model’s capability for honking sound detection, laying a solid foundation for further advancements in audio-based vehicle classification systems within the context of traffic environments.

#### The VRID dataset [31].

In pursuit of vehicle model recognition, the YOLO V9-c model is trained using the VRID dataset, which is composed of vehicle images captured by 326 high-definition cameras strategically positioned along a city bayfront, spanning 14 days of daylight hours. Characterized by resolutions ranging from 400×424 to 990×1134 pixels, the VRID dataset includes 10,000 images with a specific focus on the ten most prevalent vehicle models. Each model is depicted in 1000 unique images, as listed in Table 1. The images of each vehicle model are taken from diverse road curbs, capturing a variety of lighting conditions, scales, and poses. It is also pertinent to note that images of the same vehicle model may display a range of appearances due to differences in their visual presentations.

**Table 1:** VRID dataset summary for 10 vehicle models.

Vehicle Models	Number of Vehicles	Number of Images
Audi_A4	100	1000
Honda_Accord	100	1000
Buick_Lacrosse	100	1000
Volkswagen_Magotan	100	1000
Toyota_Corolla_I	100	1000
Toyota_Corolla_II	100	1000
Toyota_Camry	100	1000
Ford_Focus	100	1000
Nissan_Tiida	100	1000
Nissan_Sylphy	100	1000

#### The CompCars dataset [32].

The Comprehensive Cars (CompCars) dataset is a large-scale vehicle image dataset comprising two subsets: a web-nature set with 1,716 car models and a surveillance-nature subset containing images captured by real-world traffic cameras. In this work, we focus on the surveillance subset, which includes approximately 50,000 front-view vehicle images spanning 281 vehicle models. These models cover a wide range of vehicle types-such as sedans, SUVs, vans, and jeeps-offering substantially greater inter-class diversity than the sedan-only VRID dataset. Each vehicle model in the surveillance subset is represented by hundreds of images captured under varying conditions. Importantly, unlike the VRID dataset’s narrowly defined sedan categories, the broader diversity of vehicle types in the surveillance subset of the CompCars dataset presents a more coarse-grained recognition task, as vehicles often differ significantly in shape and size. This generally makes it easier for vision models to distinguish between classes.

### Performance evaluation index

The emphasis lies on recognition accuracy, thus precision and recall rate are selected as metrics to gauge recognition performance in the evaluation experiments.

Here are the definitions and formulas for recall rate (true positive rate, TPR) and precision:

Recall (True Positive Rate, TPR): It is the percentage of data samples that a machine learning model correctly identifies as belonging to the positive class out of all samples that actually belong to the positive class.

$$Recall=\frac{TruePositives}{TruePositives+FalseNegatives}.$$ (8)

Precision: It is the percentage of data samples that a machine learning model correctly identifies as belonging to the positive class out of all samples predicted to belong to the positive class.

$$Precision=\frac{TruePositives}{TruePositives+FalsePositives}.$$ (9)

These metrics are crucial for evaluating the performance of a machine learning model, especially in tasks where correctly identifying positive instances is essential.

### Evaluation experiments on vehicle model recognition process based on ECAPA-TDNN

#### Impact of background noise with different intensities on vehicle model recognition.

To investigate the impact of environmental noise on the vehicle model recognition performance using ECAPA-TDNN, an experiment was conducted. The experiment compared recognition performance under different signal-to-noise ratios (SNR) for horn audio signals mixed with environmental noise. SNRs of 10dB, 0dB, -1dB, -3dB, and -10dB were used, corresponding to horn signal powers of 10, 1, 0.8, 0.5, and 0.1 times the power of the environmental noise, respectively. Each condition included 200 randomly selected horn audio samples from the vehicle horn sound classification datasets for each vehicle model. Table 2 presents the recall rates observed under varying intensities of background noise. Table 2 illustrates that in the absence of environmental noise, vehicle model recognition achieves a mean recall rate of 99.65%. With a signal-to-noise ratio (SNR) of 10 dB, the mean recall rate remains high at 96.20%. As the SNR decreases to 0 dB, -1 dB, and -3 dB, the mean recall rates are 94.95%, 93.55%, and 92.90%, respectively. A significant decrease in mean recall rate is observed at -10 dB SNR, resulting in 75.90% accuracy. These results suggest that when the energy of the vehicle horn sound is at least half of the energy of the environmental noise, the vehicle model’s mean recall rate exceeds 92%. This indicates that the distance between the honking vehicle and the audio capture equipment has minimal impact on the vehicle model recognition performance.

**Table 2:** Vehicle model recognition recall rate under different intensities background noise.

Vehicle Models	Without Noise	With Background Noise				
		SNR=10dB	SNR=0dB	SNR=-1dB	SNR=-3dB	SNR=-10dB
Audi_A4	99.5%	97.00%	95.00%	92.00%	94.50%	94.50%
Honda_Accord	100%	97.00%	96.50%	96.50%	94.00%	96.00%
Buick_Lacrosse	99.50%	98.50%	98.50%	98.50%	97.00%	98.00%
Volkswagen_Magotan	99.50%	93.00%	94.00%	91.50%	92.00%	10.00%
Toyota_Corolla_I	99.50%	95.50%	94.50%	93.50%	96.00%	63.50%
Toyota_Corolla_II	100%	96.50%	90.50%	96.50%	96.00%	95.00%
Toyota_Camry	100%	94.00%	92.50%	95.00%	95.50%	91.50%
Ford_Focus	100%	99.50%	99.00%	99.00%	99.00%	100%
Nissan_Tiida	99.5%	95.00%	93.00%	78.00%	68.50%	18.00%
Nissan_Sylphy	99.0%	96.00%	96.00%	95.00%	96.50%	92.50%
**Mean of Recall Rate**	**99.65%**	**96.2%**	**94.95%**	**93.55%**	**92.9%**	**75.9%**

#### Impact of audio signals’ input modes on vehicle model recognition process.

During real-time recording of traffic environmental sounds, a microphone consistently captures audio signals. Prior to the vehicle model recognition process based on ECAPA-TDNN, this continuous audio signal is segmented into 2-second segments. Due to the random nature of this segmentation, it does not guarantee that each segment contains an entire horn sound. To evaluate the impact of this random segmentation on ECAPA-TDNN’s performance in vehicle model recognition, a comparative experiment was conducted. This experiment compared two audio input modes: manual audio recording mode, ensuring each segment contains a complete horn sound, and continuous audio recording mode, which simulates real-world audio acquisition where segments are randomly partitioned.

To ensure experimental rigor, multiple horn sound samples were randomly selected from the vehicle horn sound classification dataset and concatenated on a timeline with random intervals between samples. These concatenated samples were used for recognition. The performance of the vehicle model recognition process based on ECAPA-TDNN was then evaluated by continuously capturing these audio samples using a microphone. Importantly, collected audio samples were devoid of ambient noise to isolate its influence on vehicle model recognition.

The experiment recorded the recall rate and precision of vehicle model recognition across 200 occurrences of horn sounds, with detailed results presented in Table 3. Table 3 reveals that in the manual recording mode, the average precision and recall rates for vehicle model recognition are 99.47% and 99.45%, respectively. In contrast, the continuous recording mode yields average recognition precision and recall rates of 96.44% and 95.85%, respectively. This reflects a slight decrease of 3.03% in precision and 3.60% in recall rate compared to the manual recording mode. Hence, the choice of input mode minimally affects the performance of vehicle model recognition.

**Table 3:** Recall rate and precision under two input modes.

Vehicle Models	Manual Recording Mode		Continuous Recording Mode	
	Precision	Recall Rate	Precision	Recall Rate
Audi_A4	100%	100%	100%	94%
Honda_Accord	100%	99%	100%	95%
Buick_Lacrosse	100%	99.5%	99%	99%
Volkswagen_Magotan	100%	100%	100%	95%
Toyota_Corolla_I	100%	99.5%	98.4%	92%
Toyota_Corolla_II	95.69%	100%	83.76%	98%
Toyota_Camry	100%	100%	100%	95.5%
Ford_Focus	99%	99.5%	83.19%	99%
Nissan_Tiida	100%	97%	100%	97%
Nissan_Sylphy	100%	100%	100%	94%
**Mean of Index**	**99.47%**	**99.45%**	**96.44%**	**95.85%**

### Evaluation experiments on vehicle localization and model recognition based on YOLO V9-c

The YOLO V9 model is available in four variants differentiated by their parameter counts: v9-s, v9-m, v9-c, and v9-e. The YOLO V9-c model, notable for significant architectural enhancements, is specifically utilized in vehicle localization and model recognition processes. It operates with 42% fewer parameters and demands 21% less computation compared to YOLO v7, while achieving comparable accuracy.

**Fig 7:**
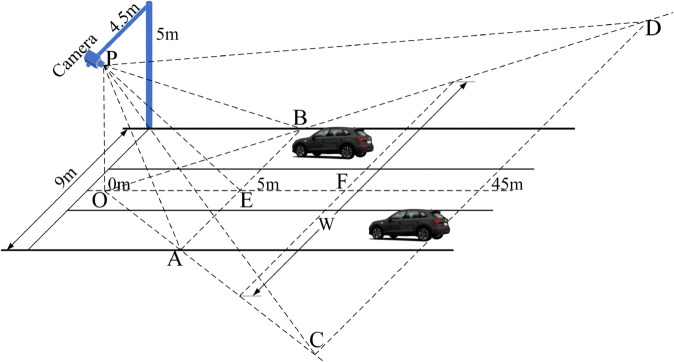
Schematic diagram of the traffic surveillance camera’s view.

To simulate the performance of vehicle localization and model recognition processes using YOLO V9-c, we consider the setup of a traffic surveillance camera as depicted in Fig 7. In Fig 7, the camera is positioned on a crossbar at a height of 5 meters, with its field of view covering the zone ABCD, spanning horizontally from 5 to 45 meters. The images captured by the camera have a resolution of 3840 × 2160 pixels. Points A and B are 9 meters apart, spanning 3 vehicle lanes, each 3 meters wide. Using the similarity of triangles depicted in Fig 7, we can derive the following equation.

$$\frac{AB}{W}=\frac{OE}{OF}.$$ (10)

Given AB=9 meters and OE=5 meters, (10) can be rewritten as follows.

$$\frac{9}{W}=\frac{5}{OF}.$$ (11)

Meanwhile, suppose a car with a width of 1.6 meters occupies w horizontal pixels in the captured image, located at point F at a distance *L* from point O. According to the perspective relationship of the camera imaging system, the following equation can be established.

$$\frac{1.6}{W}=\frac{w}{3840}.$$ (12)

By combining (11) and (12), *w* can be calculated as follows.

$$w=\frac{1.6\times 3840}{1.8L}.$$ (13)

It is evident that *w* decreases as the distance *L* increases. This relationship suggests that the distance between vehicles and the camera can be estimated from the pixel count of vehicles in the captured image. Therefore, using (13), the pixel count of vehicles at different distances *L* can be calculated. The pixel counts of vehicles at various distances are presented in Table 4. To assess the influence of distance on the performance of vehicle localization and model recognition using YOLO V9-c, 100 randomly selected images from each vehicle model in the VRID dataset were downscaled according to Table 4. The experimental outcomes are detailed in Table 5. According to Table 5, within the distance range of 5 to 25 meters, both the mean precision and mean recall rates exceed 0.99. Between 30 and 35 meters, the mean recall rate fluctuates between 0.908 and 0.945, while mean precision ranges from 0.855 to 0.951. Beyond 35 meters, there is a notable decline, with mean recall rate starting at 0.908 and mean precision at 0.855. This suggests that the vehicle localization and model recognition process using YOLO V9-c achieves significant performance at distances less than 25 meters. However, recognition performance sporadically decreases for specific vehicle models such as Toyota Corolla I, Toyota Corolla II, and Ford Focus as the distance extends from 25 to 30 meters. Furthermore, as the distance increases from 35 to 40 meters, more instances of diminished recognition performance become apparent, particularly for models like Volkswagen Magotan and Nissan Sylphy. This decrease in recognition performance is likely due to the increasing similarity in appearance of these vehicle models as the distance between the vehicle and camera increases.

**Table 4:** Pixel counts of vehicles at various distances.

Distance L (meter)	Vehicle image’s pixel count
5	683×768
10	341×384
15	228×256
20	171×192
25	137×154
30	114×128
35	98×110
40	85×96
45	76×85

**Table 5:** Performance of the vehicle localization and model recognition process based on YOLO V9-c at different distances on VRID dataset.

Vehicle models	Performance index	Distance L(meters)								
		5	10	15	20	25	30	35	40	45
Audi_A4	Precision	1	1	0.999	0.997	0.989	0.949	0.862	0.801	0.731
	Recall rate	1	1	1	1	1	0.986	0.971	0.879	0.8
Honda_Accord	Precision	1	1	1	0.999	0.994	0.994	0.976	0.911	0.92
	Recall rate	1	1	1	1	0.986	0.929	0.891	0.799	0.702
Buick_Lacrosse	Precision	1	1	0.999	0.998	1	1	0.985	0.983	0.968
	Recall rate	1	1	1	1	0.994	0.97	0.906	0.842	0.636
Volkswagen_Magotan	Precision	1	1	0.999	0.998	1	1	1	0.853	0.763
	Recall rate	1	1	1	1	1	0.959	0.867	*0.612*	*0.638*
Toyota_Corolla_I	Precision	1	0.998	0.999	1	1	1	0.978	0.974	0.925
	Recall rate	1	1	1	1	0.947	*0.683*	*0.568*	*0.465*	*0.475*
Toyota_Corolla_II	Precision	1	1	1	1	0.973	0.82	*0.482*	*0.370*	*0.286*
	Recall rate	1	0.995	0.99	0.99	0.993	0.961	0.974	0.98	0.948
Toyota_Camry	Precision	0.999	1	0.999	0.994	0.971	0.981	0.927	0.824	0.76
	Recall rate	1	1	1	1	1	0.993	0.993	0.985	0.985
Ford_Focus	Precision	1	1	0.999	0.997	0.984	*0.768*	*0.373*	*0.246*	*0.200*
	Recall rate	1	1	1	1	1	0.993	0.993	0.993	0.979
Nissan_Tiida	Precision	1	0.999	0.999	0.998	0.998	0.993	0.966	0.928	0.802
	Recall rate	1	1	1	1	1	1	0.966	0.918	0.857
Nissan_Sylphy	Precision	0.999	0.999	0.999	0.998	0.998	1	1	0.966	0.811
	Recall rate	1	1	1	1	1	0.981	0.954	*0.759*	*0.616*
Mean of precision		1	0.999	0.999	0.998	0.991	0.951	***0.855***	***0.786***	***0.716***
Mean of recall rate		1	0.999	0.999	0.999	0.992	0.945	***0.908***	***0.823***	***0.764***

Meanwhile, we conducted additional vehicle model recognition experiments on the surveillance subset of the CompCars dataset. Three experimental settings were considered:

1. a 10-class subset consisting of five popular car models overlapping with the VRID dataset and five other common vehicle models;

2. a 50-class experiment using the 50 most data-rich car models in the surveillance subset;

3. the full 281-class experiment covering all car models in the surveillance subset.

In each setting, the vehicle localization and model recognition process based on YOLOv9-c was applied to identify vehicle models at varying camera-vehicles distances, simulated by down-scaling images as described in Table 4. Table 6 presents detailed precision and recall rate results for the 10-class subset across distances ranging from 5 m to 45 m, while Table 7 summarizes the mean performance for the 50-class and 281-class experiments, presented side by side for comparison.

**Table 6:** Performance of the vehicle localization and model recognition process based on YOLO V9-c at different distances on the 10-class subset of the CompCars’s surveillance subset.

Vehicle Model	Performance index	Distance *L (meters)*								
		5	10	15	20	25	30	35	40	45
Benz_C	Precision	0.994	0.975	0.981	0.979	0.978	0.984	0.978	0.940	0.899
	Recall rate	1	1	1	1	1	1	0.998	0.964	0.964
BMW_X1	Precision	0.988	0.964	0.964	0.968	0.967	0.972	0.964	0.960	0.967
	Recall rate	1	1	1	1	0.993	0.971	0.967	0.971	0.914
Honda_Accord	Precision	0.993	0.980	0.979	0.980	0.972	0.975	0.978	0.987	1
	Recall rate	1	1	1	1	1	1	1	1	0.723
Jeep_Compass	Precision	0.996	0.986	0.988	0.987	0.986	0.981	0.985	0.988	0.999
	Recall rate	1	1	1	1	1	1	1	1	1
LAND-ROVER_Discovery	Precision	0.951	0.954	0.954	0.951	0.953	0.951	0.947	0.942	0.945
	Recall rate	0.950	0.951	0.955	0.919	0.882	0.881	0.806	0.818	0.787
Nissan_Tiida	Precision	0.967	0.954	0.953	0.953	0.955	0.954	0.954	0.953	0.948
	Recall rate	0.934	0.929	0.931	0.909	0.951	0.909	0.938	0.897	0.828
Peugeot_408	Precision	0.995	0.983	0.981	0.979	0.981	0.962	0.965	0.968	0.985
	Recall rate	1	1	1	1	1	1	1	1	1
Toyota_Camry	Precision	1	1	1	1	0.970	0.981	0.986	1	1
	Recall rate	0.995	0.989	0.987	0.974	0.974	0.979	0.974	0.971	0.728
Toyota_Corolla	Precision	0.993	0.978	0.980	0.979	0.975	0.927	0.952	0.952	0.950
	Recall rate	1	1	1	1	1	1	0.988	0.974	0.945
Volkswagen_Touareg	Precision	1	1	1	1	1	1	0.969	0.973	0.968
	Recall rate	0.962	0.959	0.959	0.959	0.938	0.949	0.919	0.865	0.824
**Mean precision**		0.988	0.977	0.978	0.978	0.974	0.969	0.968	0.966	0.966
**Mean recall rate**		0.984	0.983	0.983	0.976	0.974	0.969	0.959	0.946	0.871

**Table 7:** Performance of the AVCP method on the CompCars dataset in 50-class and 281-class settings Performance of the Vehicle Localization and Model Recognition Process Based on YOLO V9-c at Different Distances on the 50-class and 281-class Subset of the CompCars’s Surveillance Subset.

50-Class Setting									
Metric	5	10	15	20	25	30	35	40	45
Precision	0.991	0.991	0.990	0.991	0.980	0.973	0.960	0.917	0.849
Recall rate	0.992	0.992	0.989	0.981	0.980	0.973	0.930	0.876	0.796
**281-Class Setting**									
**Metric**	**5**	**10**	**15**	**20**	**25**	**30**	**35**	**40**	**45**
Precision	0.983	0.982	0.980	0.973	0.963	0.940	0.913	0.854	0.771
Recall rate	0.983	0.983	0.984	0.982	0.969	0.949	0.899	0.810	0.660

As shown in Tables 6 and 7, mean precision and recall rate on the 10-class subset remain above 0.96 up to 35 m, with recall declining moderately to about 0.87 at 45 m. Even in the full 281-class experiment, recall rate remains around 0.66 at 45 m, which is considerably higher than the corresponding performance on VRID dataset at the same distance (see Table 5). These results confirm that the proposed AVCP method achieves superior performance on the CompCars surveillance subset compared with the VRID dataset.

The superior results on the CompCars surveillance subset are largely attributable to its broader inter-class diversity. Unlike the VRID dataset, which is limited to sedans with subtle appearance differences (e.g., distinguishing between a Toyota Corolla I and Corolla II, or between a Nissan Sylphy and a Toyota Camry), the surveillance subset encompasses a wide variety of vehicle types-for instance, compact sedans, large SUVs, and jeeps-that are much easier to differentiate visually. As a result, the CompCars surveillance subset constitutes a more coarse-grained recognition task, allowing YOLOv9-c to make fewer errors even when image resolution decreases at longer distances. In contrast, the fine-grained sedan categories in the VRID dataset present a much more challenging benchmark for vehicle model recognition, where the high visual similarity among models increases the likelihood of confusion under adverse conditions.

## Conclusion and future work

In this paper, we proposed the AVCP method for honking vehicle localization and detailed its workflow. The method involves recognizing the honking vehicle’s model from captured audio signals using ECAPA-TDNN, and localizing all vehicles in captured images while identifying their models using YOLO V9. The honking vehicle is determined among recognized models with YOLO V9, matching the model identified from the audio signal, thus localizing the honking vehicle. From the experimental results, we conclude that the AVCP method for honking vehicle localization is minimally affected by the SNR of the captured audio signal, achieving a mean recall rate of up to 92.90% even at -3 dB. This indicates effective recognition of honking vehicles located at greater distances. Additionally, honking vehicles within 35 meters from the camera can be effectively located and recognized using YOLO V9. Furthermore, the continuous recording mode of audio signals has only a slight effect on the recognition performance of the vehicle model recognition process based on ECAPA-TDNN compared to manual recording mode.

The proposed AVCP method simulates human auditory and visual cooperative perception processes, offering significant advantages over techniques relying solely on sound source localization technology. This approach effectively addresses challenges common in honking vehicle detection methods using sound source localization, such as susceptibility to background noise interference, complexity of sound acquisition devices, and distance sensitivity that can reduce localization accuracy.

In our work, the performance of the proposed AVCP method for honking vehicle localization is dependent on the effectiveness of the vehicle model recognition process based on ECAPA-TDNN and the vehicle localization and model recognition process based on YOLO V9. Therefore, in future work, we plan to explore methods to reduce background noise using microphone array technology to enhance the performance of ECAPA-TDNN-based vehicle model recognition. Additionally, we will investigate applying super-resolution reconstruction methods to improve the performance of YOLO V9-based vehicle localization and model recognition.

Moreover, in scenarios where multiple vehicles of the same model appear in the captured image, with one of them honking, our future work will explore a combined approach. This approach will employ sound source localization techniques to determine the general direction of the honking vehicle within the captured image. Based on this orientation, the image will be segmented to retain the sub-image containing the honking vehicle. YOLO V9 will then be applied to this sub-image to accurately recognize and localize the honking vehicle.
